# Dyschloremia and Renal Outcomes in Critically Ill Patients With Sepsis: A Prospective Cohort Study: Dyschloremia and Renal Outcomes in Sepsis

**DOI:** 10.1155/2024/8848405

**Published:** 2024-10-01

**Authors:** Saurabh M. Thanekar, Vishal Shanbhag, Attur Ravindra Prabhu, Shankar Prasad Nagaraju, Dharshan Rangaswamy, Srinivas Vinayak Shenoy, Mohan Varadarayanahalli Bhojaraja, Indu Ramachandra Rao

**Affiliations:** ^1^ Department of Nephrology Kasturba Medical College Manipal Manipal Academy of Higher Education, Manipal 576104, Karnataka, India; ^2^ Department of Critical Care Kasturba Medical College Manipal Manipal Academy of Higher Education, Manipal 576104, Karnataka, India

**Keywords:** acute kidney injury, chloride, critical care, dyselectrolytemia, major adverse kidney events, sepsis

## Abstract

**Introduction:** Chloride is the most abundant extracellular anion; however, abnormalities of serum chloride (dyschloremia) are often overlooked. This study aimed to study the association of dyschloremia with AKI and major adverse kidney events at Day 30 (MAKE30) in critically ill patients with sepsis.

**Materials and Methods:** This prospective single-center cohort study included adult patients with sepsis admitted in a tertiary care hospital in India. Patients with advanced chronic kidney disease, requiring dialysis at admission, or with hospital stay of less than 72 h were excluded. Hyperchloremia and hypochloremia were defined as chloride levels of > 110 mEq/L and < 95 mEq/L, respectively. The primary outcome measure was MAKE30—a composite of death, need for dialysis, or sustained loss of kidney function at Day 30.

**Results:** In a cohort of 400 patients with a mean age of 60 (±15) years, AKI was seen in 301 (75.2%) and MAKE30 in 171 (42.8%). Hyperchloremia and hypochloremia were seen in 19.3% (*n* = 77) and 32.3% (*n* = 129), respectively, in the first 72 h of ICU stay. Hypochloremia, but not hyperchloremia, was independently associated with both MAKE30 (OR: 2.56, 95% CI: 1.13–5.79; *p*=0.024) and new-onset or worsening AKI (OR: 2.52, 95% CI: 1.17–5.41; *p*=0.019). There was no association between hyperchloremia and either MAKE30 (OR: 1.07, 95% CI: 0.43–2.69; *p*=0.882) or new-onset/worsening AKI (OR: 0.89, 95% CI: 0.38–2.09; *p*=0.781).

**Conclusion:** Hypochloremia, but not hyperchloremia, was associated with MAKE30 in this cohort of critically ill patients with sepsis.

**Trial Registration:** Clinical Trial Registry identifier: CTRI//2022/02/040519

## 1. Introduction

Acute kidney injury (AKI) occurs in 30% to 60% of critically ill patients and is associated with higher medical expenditure, morbidity, and death [1–5]. Despite the high incidence and adverse prognostic impact of AKI, our understanding of its etiology and risk factors remains incomplete.

One of the common electrolyte disturbances observed in the intensive care unit (ICU) is hyperchloremia, with reported incidences varying between 25% and 75% across studies [6–10]. A strong biological plausibility for hyperchloremia-associated AKI has been suggested from animal studies that have demonstrated that hyperchloremia can induce renal vasoconstriction and a reduction of glomerular filtration rate (GFR) [11–14]. Data from observational studies also suggest that hyperchloremia and the infusion of chloride-containing solutions are associated with higher in-hospital mortality and development of AKI [6, 15, 16]. Critical care physicians are increasingly turning to balanced crystalloids, such as Ringer's lactate, rather than the traditionally preferred normal saline for fluid resuscitation in the ICU in light of this evidence.

On the other hand, little attention has been paid to hypochloremia in medical literature. While hypochloremia has been described to be an independent predictor of adverse outcomes in patients with acute and chronic heart failure, its implications on patient and renal outcomes in the ICU setting remain largely unexplored [17–20].

In this prospective study, we sought to study the association between abnormalities of serum chloride levels (dyschloremia) and patient outcomes in critically ill patients with sepsis.

## 2. Methods

### 2.1. Aims

The aim of this study was to assess the effect of hyper- and hypochloremia on major adverse kidney events (MAKE30) and new-onset or worsening AKI in a cohort of critically ill patients with sepsis admitted in the medical ICUs.

### 2.2. Study Design and Setting

This was a prospective observational study conducted at a tertiary care hospital in south India between June 1, 2022, and March 31, 2023. Institutional ethics committee clearance was obtained (IEC no. 478/2021).

### 2.3. Study Participants

Patients admitted in the medical ICUs during the study period were screened for eligibility and those satisfying the inclusion and exclusion criteria as mentioned below were enrolled in the study.

Inclusion criteria were defined as follows:

• Aged 18 years or more
• Diagnosis of sepsis, as defined by the Sepsis-3 guidelines, that is, suspected/documented infection with an increase of ≥ 2 points of the Sequential Organ Failure Assessment (SOFA) score over baseline, with/without septic shock (defined as sepsis AND need for vasopressor therapy to maintain mean arterial pressure at or above 65 mm Hg with serum lactate > 2 mmol/L despite fluid resuscitation) [21–23]

Exclusion criteria were defined as follows:

• Baseline estimated glomerular filtration rate (eGFR) of < 15 mL/min/1.73 m^2^
• AKI requiring dialysis at admission
• Transferred patients with no data on intravenous fluids (type and volume) received during the first 72 h of ICU stay
• Hospital stay of less than 72 h

### 2.4. Study Procedure

After obtaining informed consent, baseline demographic, clinical, and laboratory data, including SOFA scores, were recorded. Where baseline creatinine was unavailable, it was back-calculated as per the Kidney Disease Improving Global Outcomes (KDIGO) guideline recommendations using the MDRD formula [24]. The type and quantity of replacement and maintenance intravenous fluids administered to the patient (as determined by the treating physician) were noted by the investigator. Urine output and daily net fluid balance during the first 72 h of admission were also noted. Daily chloride levels (along with arterial pH and bicarbonate) were noted from the arterial blood gas reports for the first 72 h of ICU stay. In patients where more than one blood gas reports (and therefore more than one report of chloride levels) were available, time-weighted mean chloride values over 24 h (twm_24_) were calculated as follows [25]:

(1) $$\begin{array}{c} {\text{twm}}_{24}\text{ of chloride}=\frac{\left(C1T1+C2T2+\ldots +\text{CnTn}\right)}{24}, \end{array}$$

where Cn = average chloride concentration between two consecutive time points of sampling and Tn = time (in hours) between two consecutive samples.

Chloride values of > 110 mEq/L were considered as hyperchloremia, 95–110 mEq/L were considered as normal, and < 95 mEq/L as hypochloremia. AKI was defined and staged as per the 2012 KDIGO guidelines [24]. The need for dialysis, the modality, and the dialysis dose were determined by the treating nephrologist, and details were noted by the study investigator. Patients were followed up till Day 30 for occurrence of study outcomes which were as follows (see Table 1).

### 2.5. Statistical Analysis

Assuming that 35% of the cohort would have dyschloremia, with an absolute precision of 5% and 95% confidence, and an attrition rate of 15%, we planned to recruit 400 patients. Statistical analysis was done using Jamovi version 2.3.21. Normally distributed continuous variables were described using mean and standard deviations, while median and interquartile ranges were used for non-normally distributed continuous variables. Categorical data were described as frequencies and percentages. Logistic regression was used to study the association between hyper- and hypochloremia and outcome measures. Multivariable logistic regression was done to adjust for potential confounding. The variables studied were age, gender, diabetes mellitus, chronic kidney disease, baseline SOFA score, serum albumin, procalcitonin, use of normal saline for resuscitation, cumulative fluid balance at 72 h of ICU stay, and serum sodium status (where hyponatremia, normonatremia, and hypernatremia were defined as serum sodium < 135 meq/L, 135–145 meq/L, and > 145 meq/L, respectively). Results were reported as odds ratio with 95% confidence interval. A *p* value of < 0.05 was considered to indicate statistical significance.

## 3. Results

A total of 400 consecutive eligible patients were included in the study. The baseline characteristics are described in detail in Table 2.

The mean chloride at admission was 99.5 ± 8.0 mEq/L. Hyperchloremia was observed in 30 (7.5%) at admission and in 77 (19.3%) in the first 72 h of admission. Hyperchloremic metabolic acidosis occurred in 21 patients (5.25%). Hypochloremia was observed in 102 (25.5%) at admission and in 129 (32.3%) in the first 72 h of admission.

AKI was seen in 301 (75.3%) patients overall, with Stage 1 AKI in 73 (24.3%), Stage 2 in 51 (16.9%), and Stage 3 AKI in 177 (58.8%). New-onset or worsening AKI after admission occurred in 172 (43.0%). MAKE30 occurred in 171 (42.8%), with 121 (30.3%) requiring initiation of hemodialysis, persistent renal dysfunction in 11 (2.8%) patients, and mortality in 90 (22.5%) patients.

Hypochloremia occurring in the first 72 h of ICU stay (adjusted OR: 2.56, 95% CI: 1.13–5.79; *p*=0.024), but not hyperchloremia (adjusted OR: 1.07, 95% CI: 0.43–2.69; *p*=0.882) was significantly associated with MAKE30 on multivariable logistic regression analysis (Table 3). Hypochloremia was also associated with new-onset or worsening AKI (adjusted OR: 2.52, 95% CI: 1.17–5.41; *p*=0.019), while hyperchloremia was not (adjusted OR: 0.89, 95% CI: 0.38–2.09; *p*=0.781) (Table 4). There was no association between the use of normal saline for resuscitation and MAKE30 or new-onset/worsening AKI.

SOFA score showed a significant association with both new-onset or worsening AKI (adjusted OR: 1.24, 95% CI: 1.10–1.39; *p* < 0.001) and MAKE30 (adjusted OR: 1.39, 95% CI: 1.22–1.58; *p* < 0.001). Serum albumin was associated with MAKE30 (adjusted OR: 0.51, 95% CI: 0.31–0.85; *p*=0.010), but not new-onset or worsening AKI (adjusted OR: 1.12, 95% CI: 0.75–1.91; *p*=0.438).

## 4. Discussion

In this prospective study of 400 critically ill patients with sepsis, 7.5% had hyperchloremia at admission and 19.5% went on to develop hyperchloremia in the first 72 h of ICU stay. This is consistent with a recent retrospective study by Shao et al. that also found hyperchloremia occurred in 6% of patients at baseline and increased to 20% during ICU stay [27]. This can be explained by the fact that nearly half the patients in our study received normal saline for fluid resuscitation. However, it is important to note that hyperchloremic metabolic acidosis was seen in only 5% of the cohort, thus suggesting that the risk of metabolic acidosis due to normal saline may possibly have been overstated in medical literature. On the other hand, the median fluid resuscitation volume was only 1101 mL, and so this could explain the low incidence of hyperchloremic metabolic acidosis seen in this study.

Hypochloremia also increased from 25% at admission to 32.3% during the first 72 h of hospital stay. This is a somewhat surprising finding, since given the fact that a majority of patients had received chloride-rich fluids for resuscitation, one would expect the proportion of patients with hypochloremia to be lower after the first 72 h of stay. A combination of factors such as use of diuretics, gastrointestinal loss of chloride, heart failure, adrenal insufficiency, or impaired renal chloride reabsorption may account for this finding [28].

AKI occurred in 75%, with new-onset or worsening AKI in 43%. While there was no association between hyperchloremia and new-onset or worsening AKI, hypochloremia was associated with two-fold higher odds (Table 4). Similarly, Shao et al. too reported that hypochloremia, but not hyperchloremia, was associated with development of AKI, with 70% higher odds of AKI in critically ill patients with baseline serum chloride of ≤ 94 mmol/L [27]. A retrospective study by Yessayan et al. also found that although hyperchloremia occurred in a third of patients, it was not independently associated with the risk of AKI (OR: 0.80, 95% CI: 0.51–1.25; *p*=0.33) [6]. In contrast, other studies have shown a significant association between hyperchloremia and AKI [15, 29].

MAKE30 occurred in 43% of the cohort (or in 56.8% of those with AKI). The use of major adverse kidney events (MAKE) has been recommended by the National Institute of Diabetes and Digestive and Kidney Diseases (NIDDK) workgroup for clinical trials on AKI as a relevant patient-centered outcome [26]. MAKE may be determined at Day 30 (MAKE30), Day 60 (MAKE60), or Day 90 (MAKE90); however, MAKE30 is the most commonly used since it is easy to measure and attrition is lower. Although originally proposed as an outcome measure for AKI trials, MAKE30 has since been increasingly used in observational studies as well, although one important limitation is that mortality may not always be attributable to the kidney [30, 31]. The incidence of MAKE30 has been reported to be between 16% and 28% in medical literature, which is much lower than that observed in our study [21, 30, 31]. A possible explanation for this is that while our study included only critically ill patients with sepsis, other studies have included a heterogonous ICU population. While a retrospective study by Mele et al. also only included critically ill patients with sepsis, this study defined sepsis as per ICD-10 codes, while we used the Sepsis-3 guidelines [21, 31]. Moreover, other factors such as different demographics (ethnicity, gender and age distribution, comorbidities), baseline renal function, or illness severity could also have affected the risk of MAKE30.

We found that, while hyperchloremia was not significantly associated with MAKE30, after adjusting for the effect of potential confounders, hypochloremia was associated with two-fold higher odds of MAKE30 (95% CI: 1.13–5.79; *p*=0.024). Our results are consistent with the findings of a recent retrospective study by Zhu et al., who reported that hypochloremia (but not hyperchloremia) was significantly associated with in-hospital mortality (adjusted HR: 1.46, 95% CI: 1.20–1.80; *p*=0.0003) in critically ill patients with AKI [32]. Similarly, Shao et al. also reported that hypochloremia was an independent predictor of in-hospital mortality (OR: 2.13, 95% CI: 1.52–3.00), while hyperchloremia was not (OR: 1.01, 95% CI: 0.54–1.79) [27]. The authors also reported that those with hyperchloremia had a longer length of hospital and ICU stay compared to those with hypo- and normochloremia. Kee et al. also found that, although both hyper- and hypochloremia were associated with noncomplete renal recovery in survivors of dialysis-dependent AKI, hypochloremia was associated with a much higher risk (OR: 5.12 vs. 2.53) [33]. The authors also reported that only hypochloremia was associated with a risk of persistent dialysis-dependent state (OR: 2.74; 95% CI: 1.19–6.32).

It is noteworthy that the incidence of hypochloremia was higher than hyperchloremia in this cohort. Similar results have also been reported in a previous study [27]. Taken together with the available evidence, our findings suggest that while hyperchloremia per se may have little or no impact on patient and renal outcomes, hypochloremia is a common but overlooked electrolyte abnormality in the ICU that may be associated with adverse clinical outcomes. There is a need for further prospective studies to confirm these findings. The underlying mechanisms by which hypochloremia may impact renal and patient outcomes in critically ill patients with sepsis are also currently unknown and need to be explored. Notably, low chloride was associated with adverse kidney outcomes even after adjusting for serum sodium in our cohort, suggesting a mechanism other than volume loss.

Although mechanistic data are lacking and the generalizability of our findings may be limited since it was a single-center study, to the best of our knowledge, this is the first prospective study that evaluated the association of dyschloremia with MAKE30. The other strengths lie in its relatively homogenous study population and the multivariate analysis, which allowed for comprehensive evaluation of potential confounders.

## 5. Conclusions

Dyschloremia is a common finding in the ICU and occurs in up to 50% of critically ill patients with sepsis during the first 72 h of ICU stay, with hypochloremia being more common than hyperchloremia. Hypochloremia, but not hyperchloremia, is independently associated with adverse clinical outcomes. Further prospective studies are warranted to confirm these findings and to elucidate the mechanisms underlying this relationship.

## Figures and Tables

**Table 1:** Study outcomes.

Primary outcome	Major adverse kidney outcomes within 30 days (MAKE30) (composite of death, dialysis, or sustained loss of kidney function—defined as a creatinine value ≥ 200% of baseline)—assessed at 30 days from ICU admission [26]
Secondary outcomes	New-onset acute kidney injury (defined as AKI occurring after ICU admission) OR worsening of AKI (defined as change in AKI stage after ICU admission)

**Table 2:** Baseline characteristics of the study population.

**Characteristics**	**Total (n = 400)**
Age, years (mean ± SD)	60 ± 14.5
Gender	
Male, *n* (%)	257 (64.2)
Comorbidities	
Hypertension, *n* (%)	211 (52.8)
Diabetes mellitus, *n* (%)	185 (46.2)
CKD, *n* (%)	30 (7.5)
COPD, *n* (%)	91 (22.7)
IHD, *n* (%)	58 (14.5)
CLD, *n* (%)	14 (3.5)
Malignancy, *n* (%)	8 (2)
Source of sepsis	
Lung, *n* (%)	210 (52.5)
Tropical infection, *n* (%)	59 (14.8)
Urine, *n* (%)	47 (11.8)
Other, *n* (%)	84 (20.9)
SOFA score at admission (median ± IQR)	6.0 (3.6–8.0)
SBP at admission, mmHg (mean ± SD)	123.6 ± 26.4
DBP at admission, mmHg (mean ± SD)	74.1 ± 15.4
Inotropic support, *n* (%)	138 (34.5)
Mechanical ventilation, *n* (%)	140 (35)
Hb, g/dL (mean ± SD)	11.8 ± 2.8
TLC, mm^3^ (median, IQR)	12,700 (7900–17200)
Platelet, mm^3^ (median, IQR)	229,000 (121,000–317000)
Urea, mg/dL (median, IQR)	53.0 (30.0–90.7)
Creatinine, mg/dL (median, IQR)	1.5 (1.0–3.0)
eGFR, mL/min/1.73 m^2^ (median, IQR)	46.5 (18.0–76.3)
Chloride, mEq/L (mean ± SD)	99.5 ± 8.0
Sodium, mEq/L (mean ± SD)	134 ± 7.1
Bicarbonate, mEq/L (mean ± SD)	21.8 ± 6.0
Lactate (median, IQR)	16.4 (10.8–25.5)
Albumin, g/dL (mean ± SD)	3.3 ± 0.7
Procalcitonin (median, IQR)	4.1 (1.1–12.7)
C-reactive protein (median, IQR)	108 (31.8–207)
Type of fluid, *n* (%)	
Normal saline	190 (47.5)
Ringer lactate	37 (9.3)
Others	68 (17.0)
None	105 (26.2)
Volume of resuscitation fluid, mL (median, IQR)	1101 (726–1776)

Abbreviations: CKD, chronic kidney disease; CLD, chronic liver disease; COPD, chronic obstructive pulmonary disease; DBP, diastolic blood pressure; eGFR, estimated glomerular filtration rate; Hb, hemoglobin; IHD, ischemic heart disease; IQR, interquartile range; SBP, systolic blood pressure; SD, standard deviation; SOFA, Sequential Organ Failure Assessment; TLC, total leucocyte count.

**Table 3:** Factors predictive of MAKE30.

**Covariate**	**Univariate analysis**		**Multivariable analysis**	
	**Unadjusted odds ratio (95% CI)**	**p** **value**	**Adjusted odds ratio (95% CI)**	**p** **value**
Age	0.98 (0.97–1.00)	0.062	0.99 (0.97–1.02)	0.547
Male gender	1.15 (0.76–1.74)	0.509	—	—
Diabetes mellitus	0.72 (0.48–1.07)	0.102	0.88 (0.43–1.79)	0.717
CKD	1.19 (0.56–2.50)	0.652	—	—
Serum chloride				
Normal	Ref.	—	Ref.	—
Hyperchloremia	1.60 (0.87–2.93)	0.131	1.072 (0.43–2.69)	0.882
Hypochloremia	1.45 (0.85–2.47)	0.145	2.56 (1.13–5.79)	**0.024**
Serum sodium				
Normal	Ref.	—	Ref.	—
Hypernatremia	1.40 (0.47–4.22)	0.549	0.93 (0.28–3.06)	0.902
Hyponatremia	2.04 (1.33–3.13)	0.001	1.80 (0.78–4.18)	0.169
SOFA score	1.42 (1.31–1.55)	< 0.001	1.39 (1.22–1.58)	**< 0.001**
Normal saline for resuscitation	0.86 (0.52–1.42)	0.565	—	—
Cumulative fluid balance	1.00 (1.00–1.00)	0.006	1.00 (0.99–1.00)	0.319
Albumin	0.44 (0.32–0.60)	< 0.001	0.51 (0.31–0.85)	**0.010**
Procalcitonin	1.00 (0.99–1.01)	0.121	1.00 (0.99–1.01)	0.713

Abbreviations: CI, confidence interval; CKD, chronic kidney disease; SOFA, Sequential Organ Failure Assessment.

*p* values of less than 0.05 have been highlighted in bold for emphasis.

**Table 4:** Factors predictive of new-onset/worsening AKI.

**Covariate**	**Univariate analysis**		**Multivariable analysis**	
	**Unadjusted odds ratio (95% CI)**	**p** **value**	**Adjusted odds ratio (95% CI)**	**p** **value**
Age	0.99 (0.98–1.00)	0.174	0.99 (0.97–1.01)	0.348
Male gender	1.53 (1.00–2.33)	0.046	1.16 (0.83–3.09)	0.159
Diabetes mellitus	0.76 (0.51–1.14)	0.185	1.12 (0.58–2.18)	0.734
CKD	0.88 (0.41–1.87)	0.730	—	—
Serum chloride				
Normal	Ref.	—	Ref.	—
Hyperchloremia	1.00 (0.55–1.82)	0.989	0.89 (0.38–2.09)	0.781
Hypochloremia	1.49 (0.90–2.48)	0.123	2.52 (1.17–5.41)	**0.019**
Serum sodium				
Normal	Ref.	—	Ref.	—
Hypernatremia	1.65 (0.56–4.91)	0.368	0.98 (0.18–5.41)	0.990
Hyponatremia	1.58 (1.04–2.41)	0.034	1.33 (0.67–2.70)	0.429
SOFA score	1.26 (1.18–1.35)	< 0.001	1.24 (1.10–1.39)	**<** **0.001**
Normal saline for resuscitation	0.77 (0.47–1.28)	0.316	—	—
Cumulative fluid balance	1.00 (1.00–1.00)	0.012	1.00 (0.99–1.00)	0.100
Albumin	0.77 (0.58–1.03)	0.076	1.20(0.75–1.91)	0.438
Procalcitonin	1.00 (0.99–1.01)	0.192	1.00 (0.99–1.00)	0.903

Abbreviations: CI, confidence interval; CKD, chronic kidney disease; SOFA, Sequential Organ Failure Assessment.

*p* values of less than 0.05 have been highlighted in bold for emphasis.

## Data Availability

The data used to support the findings of the study are available from the corresponding author upon request.
